# The Agonist Action of Alkylphenols on TRPA1 Relates to Their Effects on Membrane Lipid Order: Implications for TRPA1-Mediated Chemosensation

**DOI:** 10.3390/ijms22073368

**Published:** 2021-03-25

**Authors:** Justyna B. Startek, Alina Milici, Robbe Naert, Andrei Segal, Yeranddy A. Alpizar, Thomas Voets, Karel Talavera

**Affiliations:** 1 Laboratory of Ion Channel Research, Department of Cellular and Molecular Medicine, KU Leuven, 3000 Leuven, Belgium; justyna.startek@kuleuven.be (J.B.S.); milicialina13@gmail.com (A.M.); robbe.naert@kuleuven.be (R.N.); andrei.segal@kuleuven.be (A.S.); yeranddy.aguiaralpizar@uhasselt.be (Y.A.A.); thomas.voets@kuleuven.be (T.V.); 2 VIB Center for Brain & Disease Research, 3000 Leuven, Belgium

**Keywords:** TRPA1, alkylphenols, mechanosensation, membrane fluidity, Laurdan, DPH

## Abstract

The Transient Receptor Potential Ankyrin 1 cation channel (TRPA1) is a broadly-tuned chemosensor expressed in nociceptive neurons. Multiple TRPA1 agonists are chemically unrelated non-electrophilic compounds, for which the mechanisms of channel activation remain unknown. Here, we assess the hypothesis that such chemicals activate TRPA1 by inducing mechanical perturbations in the plasma membrane. We characterized the activation of mouse TRPA1 by non-electrophilic alkylphenols (APs) of different carbon chain lengths in the para position of the aromatic ring. Having discarded oxidative stress and the action of electrophilic mediators as activation mechanisms, we determined whether APs induce mechanical perturbations in the plasma membrane using dyes whose fluorescence properties change upon alteration of the lipid environment. APs activated TRPA1, with potency increasing with their lipophilicity. APs increased the generalized polarization of Laurdan fluorescence and the anisotropy of the fluorescence of 1,6-diphenyl-1,3,5-hexatriene (DPH), also according to their lipophilicity. Thus, the potency of APs for TRPA1 activation is an increasing function of their ability to induce lipid order and membrane rigidity. These results support the hypothesis that TRPA1 senses non-electrophilic compounds by detecting the mechanical alterations they produce in the plasma membrane. This may explain how structurally unrelated non-reactive compounds induce TRPA1 activation and support the role of TRPA1 as an unspecific sensor of potentially noxious compounds.

## 1. Introduction

Animal survival strongly relies on constant monitoring of the external chemical environment that allows the trigger of adequate protective reactions against potentially noxious stimuli. Chemosensation mainly occurs via the senses of smell and taste and by free nerve fibers in the mucosa and skin [1], which is crucial for the detection of environmental pollutants and plant-derived chemical irritants [2]. The interaction of such compounds with nociceptive afferent fibers of the somatosensory nervous system can produce pungent, burning, and pain sensations and inflammatory responses [3]. Several members of the Transient Receptor Potential superfamily of cation channels are expressed in sensory neurons, acting as receptors of (potentially) noxious chemical stimuli [1]. These channels are actually polymodal nociceptors, integrating the effects of chemical, thermal, and mechanical stimuli into channel opening and cation influx, which in turn modulates cellular electrical excitability and Ca^2+^-dependent signaling pathways [4,5,6]. The Ca^2+^-permeable non-selective cation channel TRPA1 is arguably the most versatile of such receptors expressed in nociceptive neurons [7], skin keratinocytes [8,9], and airway epithelial cells [10].

TRPA1 is implicated in the pathophysiology of pain and inflammation as a sensor of thermal and mechanical stimuli, but it is best known for being activated by an extremely wide variety of noxious chemicals [11,12]. For instance, TRPA1 is activated by electrophilic compounds such as allyl isothiocyanate (AITC) and cinnamaldehyde [13,14]. This type of compound is thought to act on the channel through a mechanism that involves covalent modification of cysteine and lysine residues in the N-terminal region [15,16]. However, it has been shown that truncated versions of human and mosquito TRPA1 lacking this region that can be activated by electrophiles [17,18]. Furthermore, a more recent study using mass spectrometry unveiled a complex pattern of interactions between the electrophile N-methylmaleimide (NMM) and multiple cysteine and lysine residues of human TRPA1. It was found that increasing concentrations of NMM resulted in more labeling of some cysteine residues by lower labeling of others. Therefore, it was concluded that further structural and functional studies are required to elucidate the mechanisms underlying TRPA1 activation by electrophilic compounds [19].

TRPA1 is also activated by an extremely large number of non-electrophilic chemicals [12]. The list includes, among many others, local and general anesthetics [20,21,22,23,24], dihydropyridines [25], monoterpenes [26,27], alcohols [28], alkaloids [29,30], and bacterial lipopolysaccharides [31,32]. Structure-function studies based on a chimeric approach revealed that residues in the transmembrane segment 5 are essential for activation of human and mouse TRPA1 by the non-electrophilic agonist menthol [33]. However, evidence directly demonstrating that such residues form actual binding sites is currently lacking. Recently, efforts were made to employ an algorithm predicting binding and activation efficiency of different chemicals to TRPA1. Even though the proposed screening algorithm was able to identify potential TRPA1 ligands based on their structure it was unable to discriminate between activation and inhibition activities or indicate a single binding site. Although such an in silico approach could indicate potential agonists and antagonists, further in vitro and in vivo studies are needed to confirm the biological activity and their action mechanism [34]. Thus, the mechanism underlying the activation by non-electrophiles and the lack of specificity of TRPA1 for such compounds remains elusive.

Although it cannot be fully discarded at this point, it seems unlikely that TRPA1 has binding pockets able to interact with such a wide variety of chemical structures. Alternatively, previous studies suggest a hypothesis that links the chemical activation of the channel to its mechanosensory properties. For instance, it was shown that TRPA1 is activated by trinitrophenol and by the tarantula toxin GsMTx-4, which insert preferentially in the outer leaflet of the bilayer, thereby inducing local membrane cupping [35]. Furthermore, the agonistic effects of primary and secondary alcohols positively correlate with their lipophilicity [28]. Finally, the stimulatory action of different lipopolysaccharides was shown to be related to their ability to alter the local lipid environment in the plasma membrane [31,36,37,38,39]. Noting that many non-electrophilic agonists of TRPA1 are amphiphilic, these findings suggest that the channel is activated by mechanical perturbations produced by compound insertion in the plasma membrane. In this study, we addressed this hypothesis by testing a family of alkylphenols (APs) in terms of their effects on TRPA1 and of their ability to induce mechanical perturbations in the plasma membrane, and by determining whether these properties relate to each other.

We found that APs are TRPA1 agonists, via a mechanism that is not mediated by oxidative stress, nor by covalent modification of key cysteine residues involved in the activation by electrophilic compounds. On the other hand, the potency of the APs strongly correlated with the length of their carbon chain and lipophilicity. This relation cannot be explained in terms of a model based on distinct access of the compounds to a putative membrane-buried binding site in mTRPA1. Notably, the lipophilicity of the APs also related to their ability to alter the local lipid environment in the plasma membrane, as assessed with fluorescence spectroscopy and imaging experiments with Laurdan and 1,6-diphenyl-1,3,5-hexatriene (DPH). We propose that TRPA1 activation by non-electrophilic compounds may arise from the induction of mechanical perturbations in the plasma membrane, and that the chemical detection via mechanosensation is an efficient mechanism supporting the role of TRPA1 as an unspecific sensor of potentially noxious compounds. We conclude that more research is needed to determine whether mTRPA1 can be gated by non-electrophilic compounds through direct binding and/or by the induction of physical changes in its lipid environment.

## 2. Results

### 2.1. Alkylphenols Activate mTRPA1

We tested the effects of six APs, all alkylated in the para position, with carbon chains of different lengths (4-methylphenol; 4-ethylphenol; 4-propylphenol; 4-pentylphenol; 4-heptylphenol; and 4-octylphenol, Sigma-Aldrich, Steinheim, Germany). The calculated octanol-water partition coefficient (logP) of these compounds, a proxy for their ability to insert in the plasma membrane, increases with the length of the carbon chain (Table 1).

To test the effects of APs on mouse TRPA1 we first performed intracellular Ca^2+^ imaging using the Ca^2+^ sensitive dye Fura-2 in Chinese hamster ovary (CHO) cells stably transfected with this channel (CHO-mTRPA1). 

**Figure 1:**
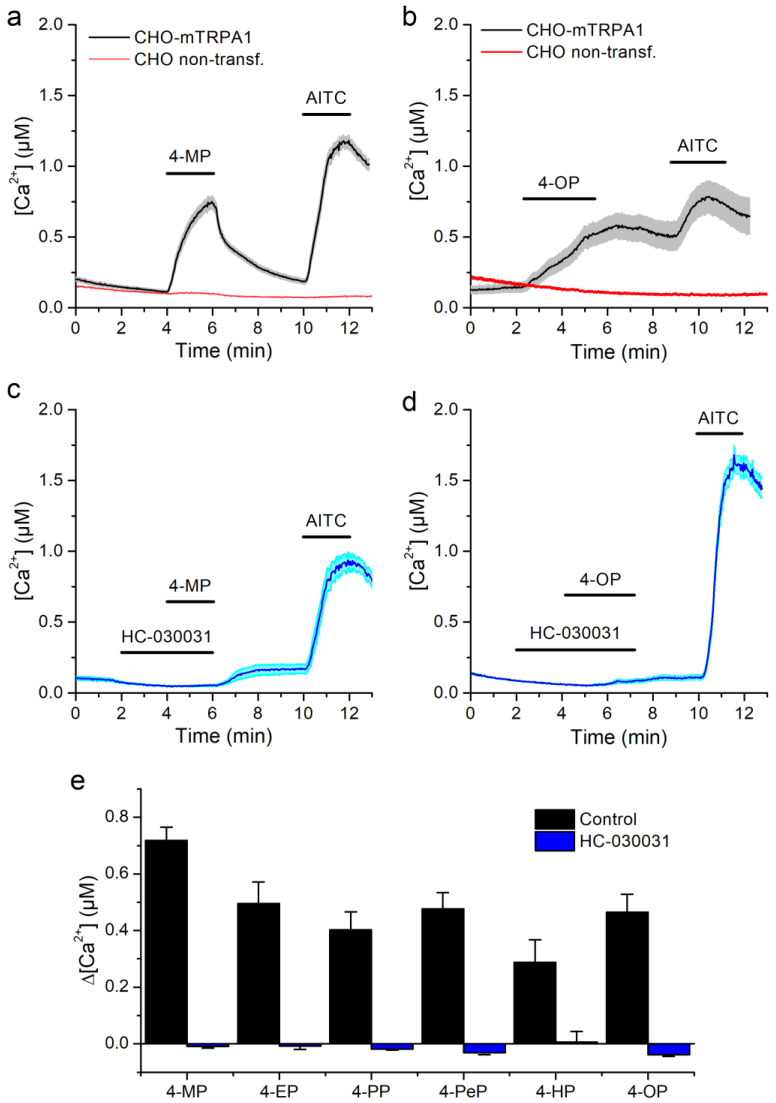
mTRPA1-mediated responses to alkylphenols. (**a**,**b**) Average traces of [Ca^2+^] recorded in CHO-mTRPA1 or non-transfected CHO cells showing the effects of 4-methylphenol (4-MP, 1300 µM, **a**) and 4-octylphenol (4-OP, 3.8 µM, **b**). (**c**,**d**) Average traces of [Ca^2+^] recorded in CHO-mTRPA1 cells showing the lack of effects of 4-methylphenol (4-MP, 1300 µM, **c**) and 4-octylphenol (4-OP, 3.8 µM, **d**) in the presence of TRPA1 inhibitor HC-030031 (50 µM). (**e**) Average [Ca^2+^] change induced by alkylphenols (APs) in the control condition and in the presence of HC-030031. APs were applied at concentrations of 1300, 110, 60, 38, 10, and 3.8 µM for 4-MP, 4-EP, 4-PP, 4-PeP, 4-HP, and 4-OP respectively. The data is represented as mean ± s.e.m. (*n* > 50).

All APs tested were able to increase in intracellular Ca^2+^ concentration [Ca^2+^], albeit at different concentrations, with higher concentrations being required for APs of shorter carbon chain (Figure 1a,c,e). All cells responding to the APs were also stimulated by the TRPA1 agonist AITC (100 µM, Sigma-Aldrich, Bornem, Belgium). In contrast, neither non-transfected CHO cells nor CHO-mTRPA1 treated with the mTRPA1 inhibitor HC-030031 (Sigma-Aldrich) [40] responded to any of the APs (Figure 1a–e).

**Figure 2:**
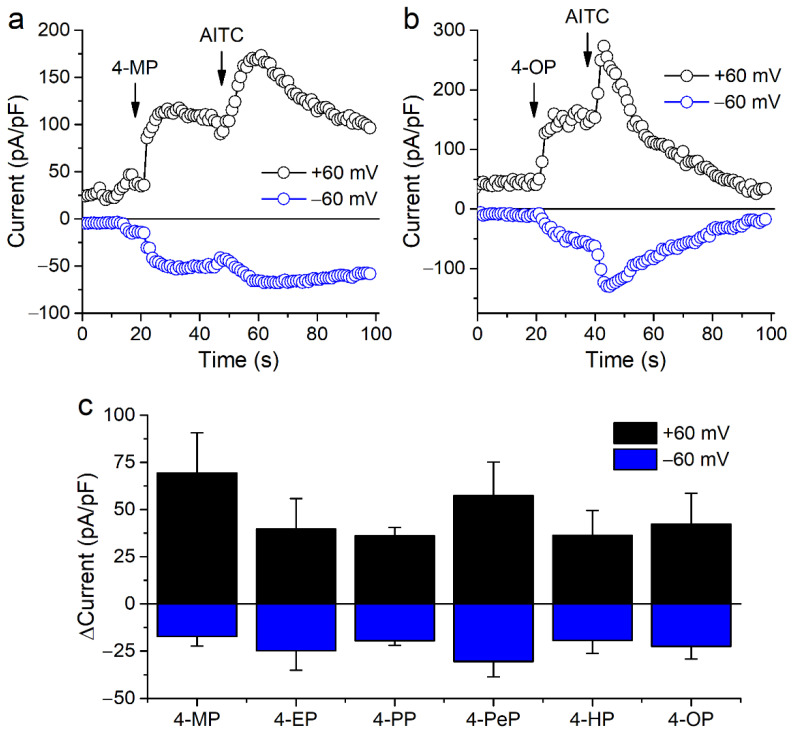
Alkylphenols stimulate mTRPA1 currents. (**a**,**b**) Time course of mTRPA1 current amplitudes measured at −60 and +60 mV. 4-methylphenol (4-MP), 4-octylphenol (4-OP) and allyl isothiocyanate (AITC; 100 µM) were applied at the time points indicated by the arrows. (**c**) Change in current densities at −60 and +60 mV induced by 4-MP (1500 µM), 4-EP (600 µM), 4-PP (100 µM), 4-PeP (200 µM), 4-HP (30 µM), 4-OP (30 µM). Data is shown as mean ± s.e.m. (*n* ≥ 4).

We further tested the effects of APs on mTRPA1 by performing whole-cell patch-clamp experiments in CHO-mTRPA1 cells. Extracellular application of APs increased inward and outward currents, and subsequent applications of AITC (100 µM) produced additional current stimulation (Figure 2a–c). As in the Ca^2+^ imaging experiments, higher concentrations of APs of shorter carbon chains were required for current stimulation.

### 2.2. Activation of mTRPA1 by Alkylphenols Is Not Mediated by Reactive Agents

**Figure 3:**
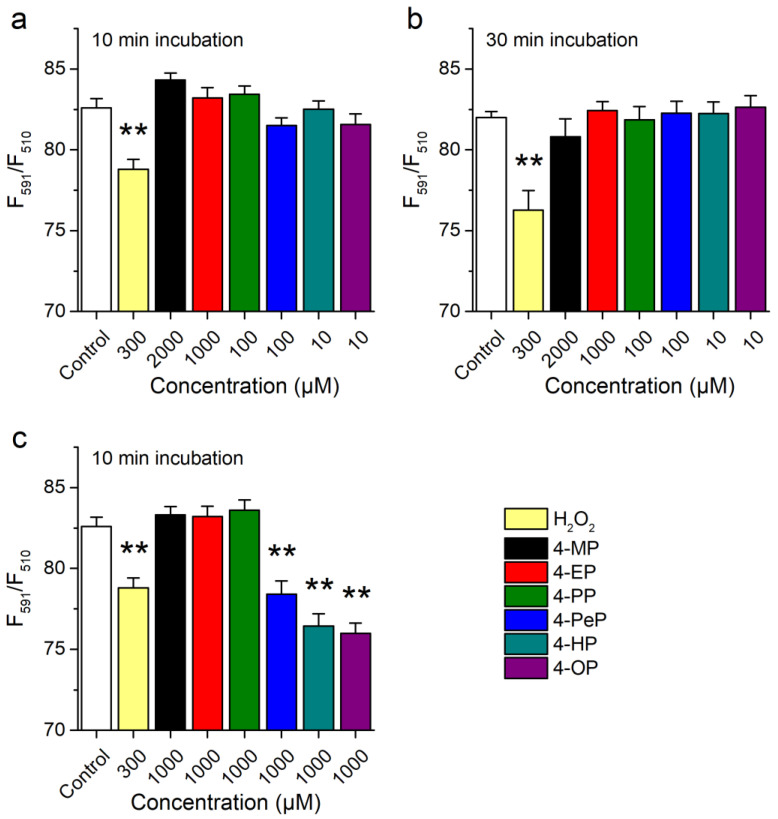
Alkylphenols do not induce lipid peroxidation at concentrations inducing mTRPA1 activation. (**a**,**b**) Ratiometric assessment of lipid peroxidation after 10 min (**a**) and 30 min (**b**) treatment with 4-MP (2000 µM), 4-EP (1000 µM), 4-PP (100 µM), 4-PeP (100 µM), 4-HP (10 µM), 4-OP (10 µM) or 300 µM H_2_O_2_. (**c**) Comparison of the effect induced by APs at a concentration of 1000 µM. Data is presented as mean ± s.e.m. from three experiments (** *p* < 0.01, two-tailed Mann-Whitney U test).

TRPA1 is activated by reactive oxygen species, such as H_2_O_2_ [12,41,42,43]. Thus, it could be envisaged that APs activate mTRPA1 by inducing oxidative stress. We assessed this possibility by testing if APs increase oxidation levels, using the Image-iT Lipid Peroxidation Kit that is based on the fluorescent reporter BODIPY 581/591 C11. The fluorescence of this reagent shifts from red to green upon oxidation, allowing for ratiometric determinations of lipid peroxidation. Treatments of CHO-mTRPA1 cells over 10 or 30 min with APs at concentrations activating mTRPA1 did not induce lipid peroxidation (Figure 3a,b). When tested at 1000 µM, only 4-PeP, 4-HP, and 4-OP induced significant lipid peroxidation (Figure 3c). As expected, H_2_O_2_ (300 µM) caused a significant oxidative stress (Figure 3c).

**Figure 4:**
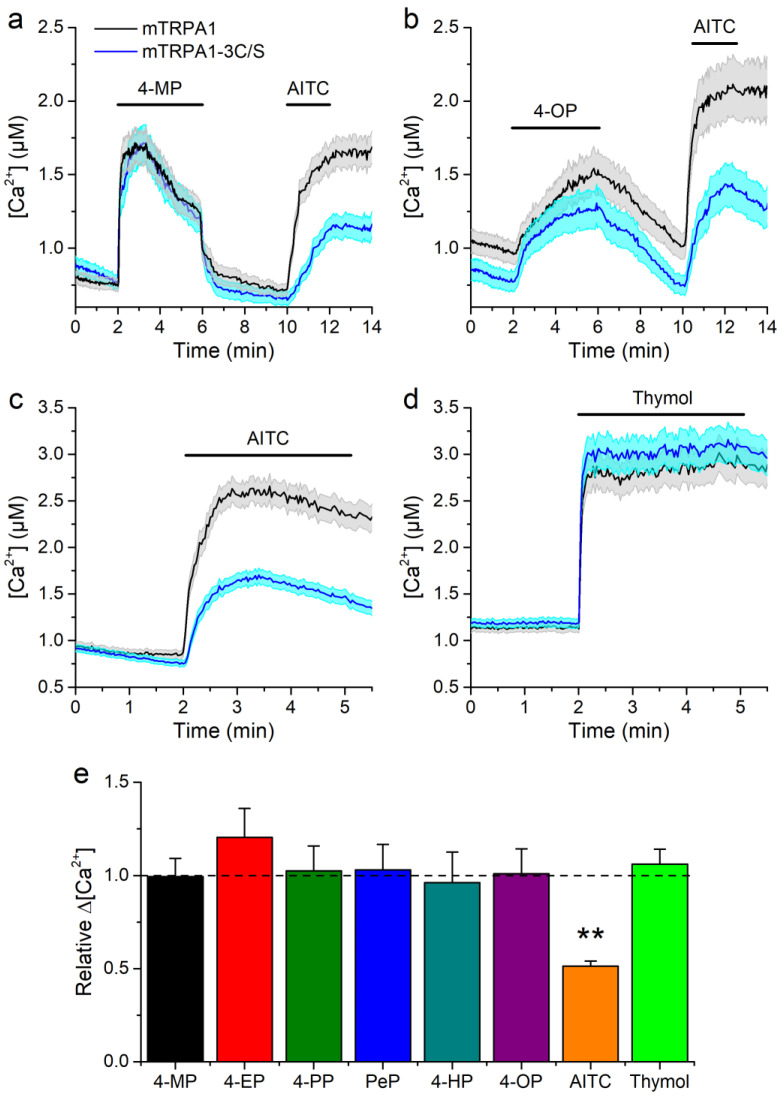
Responses to alkylphenols are not affected by mutation of mTRPA1 N-terminal essential residues. (**a**–**d**) Average traces of [Ca^2+^] levels recorded in cells transfected with the wild type mTRPA1 or the triple cysteine mutant (mTRPA1-3C/S) in the presence of (**a**,**b**) 4-methylphenol, 1300 µM (**c**,**d**) 4-octylphenol, 3.8 µM and TRPA1 agonist AITC, 100 µM. (**e**) Average [Ca^2+^] amplitude change evoked by APs in wild type and mutant mTRPA1. APs were applied at concentrations 1300, 110, 60, 38, 10, 3.8 µM for 4-MP, 4-EP, 4-PP, 4-PeP, 4-HP, 4-OP respectively, 100 µM AITC and 300 µM thymol. The error bars represent the standard error of the mean. All experiments were conducted in transfected HEK293T cells (*n* > 50) (** *p* < 0.01, two-tailed Mann-Whitney U test).

Another possibility is that APs induce the production of electrophilic compounds, which are known to activate mTRPA1. We assessed this by testing the effects of APs on an electrophile-resistant mutant channel in which residues C622, C642, and C666 are substituted to serine (mTRPA1-3C/S) [15,16,44]. The responses of this mutant to the APs were not significantly different from the response of the wild type channel (Figure 4a,b,e). As expected, the mutant channel showed reduced response to the electrophile AITC (100 μM), but unaltered response to the non-electrophilic agonist thymol (Figure 4c–e).

These results indicate that APs-induced activation does not involve oxidative stress nor the action of electrophilic intermediates. Both the intracellular Ca^2+^ imaging and patch-clamp experiments indicated that APs act as mTRPA1 agonists with different potencies. We aimed at confirming this notion by determining the concentration dependence, as we reasoned that such differences may provide insight into the mechanisms of action of these compounds on TRPA1.

### 2.3. Concentration-Dependent Effects of Alkylphenols on mTRPA1

**Figure 5:**
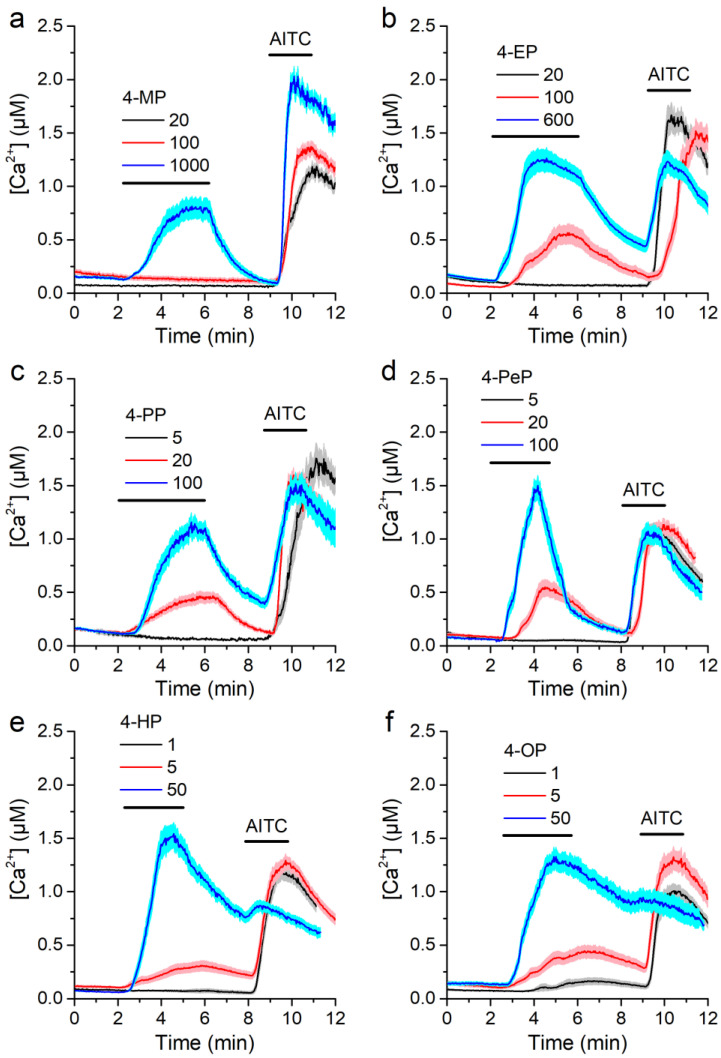
Alkylphenols activate mTRPA1 in a concentration-dependent manner. (**a**–**f**) Average traces of [Ca^2+^] recorded in CHO-mTRPA1 cells showing responses to 4-methylphenol (4-MP, **a**), 4-ethylphenol (4-EP, **b**), 4-propylphenol (4-PP, **c**), 4-penthylphenol (4-PeP, **d**), 4-heptylphenol (4-HP, **e**) and 4-octylphenol (4-OP, **f**). AITC 100 µM was applied as a positive control for mTRPA1 activation. Data from *n* > 50 cells are shown as mean ± s.e.m.

Cells were highly insensitive to 4-MP, as concentrations of 1000 µM or higher were required to elicit Ca^2+^ responses (Figure 5a). 4-EP was able to trigger responses already at 100 µM, but was ineffective at 20 µM (Figure 5b). 4-PP and 4-PeP were effective at 20 µM, but not at 5 µM (Figure 5c,d). 4-HP induced responses at 5 µM, but not at 1 µM (Figure 5e), whereas 4-OP did trigger responses at this concentration (Figure 5f).

**Figure 6:**
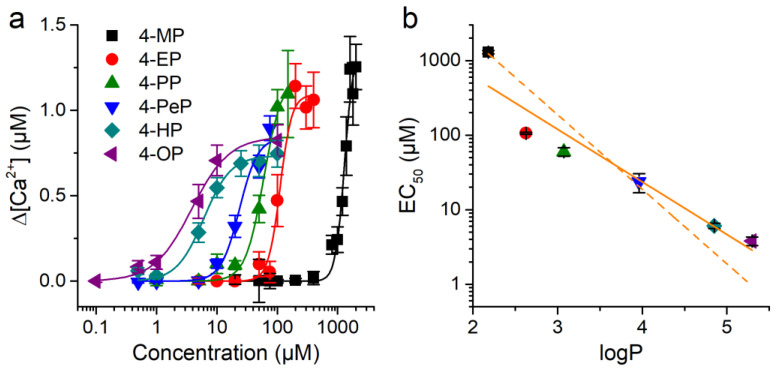
mTRPA1 activation by alkylphenols correlates with compound lipophilicity. (**a**) Concentration-dependent responses of mTRPA1 to APs from which compounds EC_50_ values were determined. Data from > 50 cells shown as mean ± s.e.m. (**b**) Relationship between the EC_50_ for mTRPA1 activation and logP across APs. The lines represent fittings with linear functions with slope constrained to −1 (dashed) or the best fit with unconstrained slope (continuous) (see text for more details).

The plot of the average amplitude of the responses of CHO-mTRPA1 cells as a function of the concentration of the APs corroborates that the effects were concentration-dependent and illustrates difference in sensitivity of mTRPA1 for these compounds (Figure 6a). Each of these experimental data sets were fit with Hill functions, yielding the corresponding values for effective concentration, EC_50_. The EC_50_ is a decreasing function of logP of the compounds (Figure 6b).

Because of the different lipophilicities of the APs, it can be hypothesized that their distinct potencies are solely due to the different accessibilities of these compounds to a hypothetical binding site in the channel. This scenario implies two conditions: (1) that the effective concentration reaching the binding site is determined by how much the AP partitions in the plasma membrane; and (2) that the interactions with the binding site is the same for all APs.

Channel activation (estimated from the increase in [*Ca*^2+^]) is a function of the alkylphenol (AP) concentration reaching the binding site ([*AP*]*_B_*):

$$\Delta \left[Ca^{2+}\right]=\frac{\Delta {\left[Ca^{2+}\right]}_{Max}}{1+{\left(\frac{K_{d}}{{\left[AP\right]}_{B}}\right)}^{H}}$$

where *K_d_* is the equilibrium constant of binding, *H* is the Hill coefficient and $\Delta {\left[Ca^{2+}\right]}_{Max}$ is the maximal effect (efficacy).

The concentration of the APs at the binding site, [*AP*]*_B_*, is given by:

$${\left[AP\right]}_{B}=P_{AP}\cdot {\left[AP\right]}_{E}$$

where *P_AP_* is the partition coefficient and [*AP*]*_E_* is the AP concentration in the extracellular solution.

Thus, the dose responses for the APs can be written as:

$$\Delta \left[Ca^{2+}\right]=\frac{\Delta {\left[Ca^{2+}\right]}_{Max}}{1+{\left(\frac{K_{d}}{P_{AP}\cdot {\left[AP\right]}_{E}}\right)}^{H}}$$

This equation can be re-written as:

$$\Delta \left[Ca^{2+}\right]=\frac{\Delta {\left[Ca^{2+}\right]}_{Max}}{1+{\left(\frac{EC_{50}^{Exp}}{{\left[AP\right]}_{E}}\right)}^{H}}$$

where $EC_{50}^{Exp}=K_{d}/P_{AP}$ is the effective concentration for channel activation determined experimentally. Thus,

$$\log\left(EC_{50}^{Exp}\right)=\log\left(K_{d}\right)-\log\left(P_{AP}\right)$$

This equation implies that the logarithm of the effective concentration for channel activation determined experimentally, $\log\left(EC_{50}^{Exp}\right)$, is a linear function of log(*P_AP_*), with slope-1. The fit of the experimental data with this equation yields an unsatisfactory result, with up to 4-fold deviations from the *EC*_50_ values of 4-EP, 4-PP, 4-HP, and 4-OP (Figure 2b, dashed line). The coefficient of determination of this fit is R^2^ = 0.75. This value is lower than the R^2^ value of the best linear fit (0.91), which yields a slope of −0.70 ± 0.11 (Figure 6b, continuous line). In addition, this simple model does not account for the distinct efficacies and Hill coefficients obtained from the fits of the concentration dependencies of the different APs (Figure 6a; data not shown). Taken together, this demonstrates that the differences in lipophilicity across APs are not sufficient to explain the distinct effects of these compounds on mTRPA1.

It might be possible that more complex models based on putative APs binding to mTRPA1 fit better our data. However, the finding that the *EC*_50_ of APs to activate mTRPA1 decreases with their ability to partition into the plasma membrane may point to the alternative hypothesis that mTRPA1 activation by these compounds is related to the induction of mechanical perturbations in the plasma membrane. To test this, we assessed the ability of APs to alter the lipid environment, using fluorescence-based methods to evaluate membrane order, fluidity, and integrity.

### 2.4. Alkylphenols Increase Membrane Order

Firstly, we used Laurdan as a probe serving to report on changes in lipid order that may be induced by the partition of APs in the membrane CHO-mTRPA1 cells. This fluorophore displays a red-shifted emission when in contact with water molecules that penetrate the membrane upon an increase in local lipid disorder [45]. Changes in the membrane properties can be detected by measuring changes in the Generalized Polarization (GP) parameter determined at 340 nm (ΔexGP^340^) [45,46].

**Figure 7:**
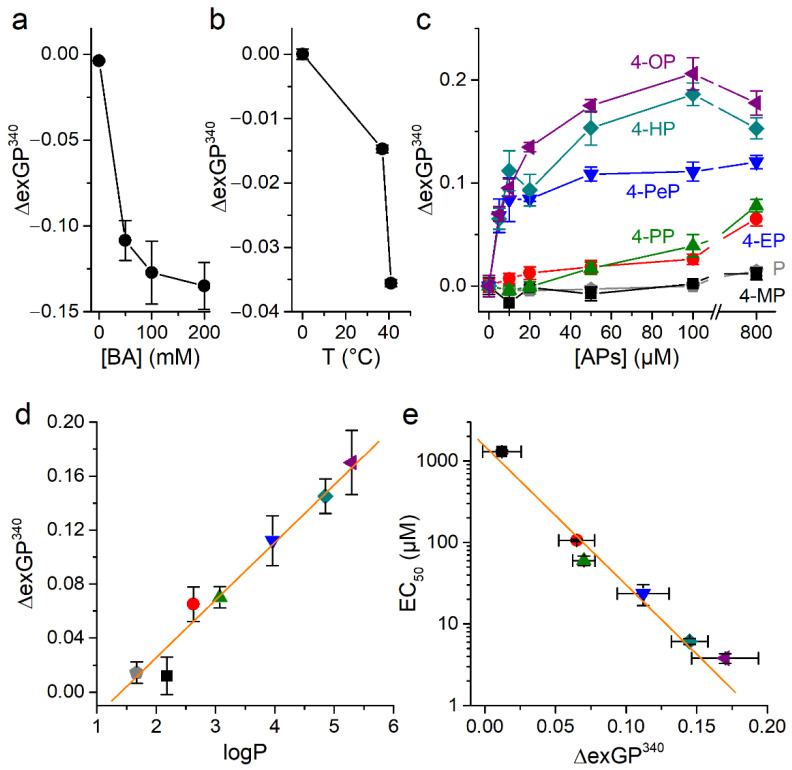
Phenol and alkylphenols induce changes of membrane fluidity. (**a**) Changes in exGP^340^ induced by the membrane fluidizing compound benzyl alcohol (BA, 0–200 mM). (**b**) Changes in exGP^340^ induced by changing temperature. (**c**) Concentration-dependent changes in exGP^340^ induced by of phenol and APs. All experiments were conducted in CHO-TRPA1 cells stained with 1.5 µM Laurdan and measured at T = 37 °C. The data is shown as mean ± s.e.m. from at least three experiments performed in triplicate. (**d**) Relationship between the ΔexGP^340^ attributed to the rise of membrane order and the partition coefficient of phenol and APs (800 µM). The orange line represents the fit with a linear function with a slope of 0.043 ± 0.003 (Pearson’s correlation coefficient 0.98, R^2^ = 0.97). (**e**) Relationship between APs EC_50_ value for TRPA1 activation and change in exGP^340^ induced by application of the APs (800 µM). The orange line represents the fit with a linear function with a slope of −17.0 ± 1.3 µM (Pearson’s correlation coefficient −0.99, R^2^ = 0.98).

In control experiments, we tested the effects of treating cells with benzyl alcohol (BA), a known bilayer disordering agent [47,48]. As expected, BA caused a concentration dependent decrease in exGP^340^ (Figure 7a). On the other hand, cooling induced the expected increase in exGP^340^, which is consistent with an increase in the membrane order (Figure 7b).

Treatment with each AP tested increased exGP^340^ in a concentration-dependent manner (Figure 7c), indicating that these compounds induce membrane ordering. However, the effectiveness to induce this effect strongly varied across compounds, with those with larger carbon chain (higher lipophilicity) being the most effective. For instance, 4-PeP, 4-HP, and 4-OP induced changes in exGP^340^ at the lowest concentration tested (5 µM, with *p* = 0.017, 0.01, 0.01, respectively, U test), whereas 4-PP and 4-EP required to be applied at 50 and 400 µM, respectively, for increases in this parameter to be significant (*p* = 0.01 and 0.04, respectively, U test). Furthermore, 4-MP and the non-alkylated form, phenol, only induced a significant increase in exGP^340^ when tested above 800 µM. In addition, the effects of 4-PeP, 4-HP, and 4-OP reached saturation for concentrations above ~50 µM, whereas this was not observed for any of the other compounds for concentrations up to 800 µM. The effects of APs on exGP^340^ can be represented as a function of the lipophilicity of the compounds (Figure 7d). This shows that the ability of APs to alter membrane order is a linear function of the lipophilicity, and that this dependency was concentration dependent.

To determine how the potency of APs for mTRPA1 activation relates to their ability to induce membrane order we plotted the EC_50_ values obtained in Ca^2+^ imaging versus the effects of the compounds on exGP^340^ (Figure 7e). These representations show a direct linear relation between induced by APs changes in the membrane order and their ability to activate mTRPA1.

### 2.5. Alkylphenols Increase Membrane Rigidity

**Figure 8:**
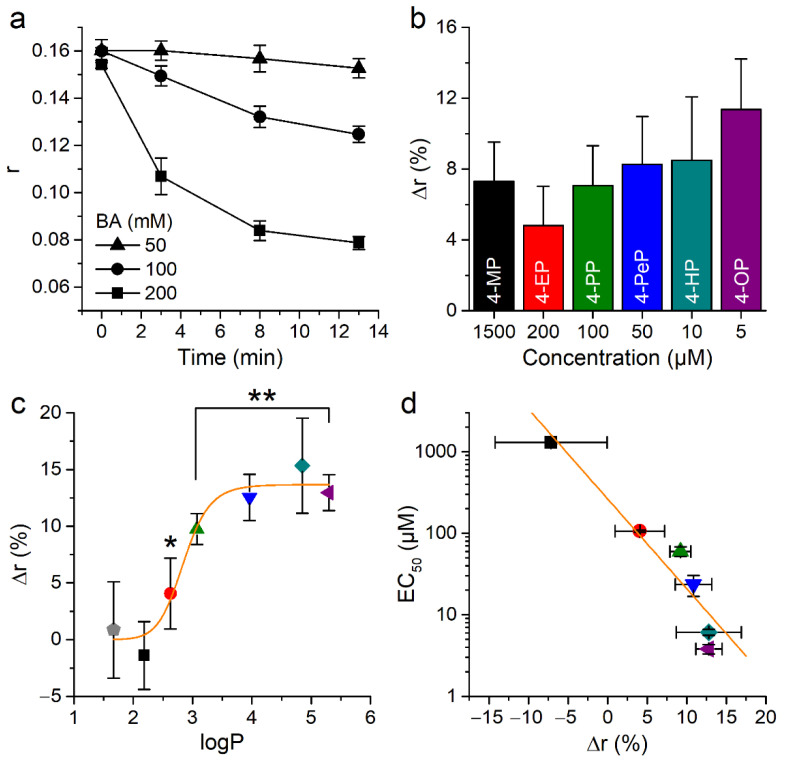
Effect of alkylphenols on the anisotropy of 1,6-diphenyl-1,3,5-hexatriene (DPH) fluorescence. (**a**) Time course of anisotropy changes induced by application of 50, 100, or 200 mM of BA. (**b**) Change in anisotropy (after 3 min pre-treatment) caused by near to EC_50_ for mTRPA1 concentrations of APs. All experiments were conducted in CHO-mTRPA1 cells stained with 10 µM DPH and measured at T = 37 °C using FlexStation III. The data is shown as mean ± s.e.m. from at least three experiments. (**c**) Relationship between changes in membrane fluidity induced by the phenol and its derivatives (all at 10 µM) and the calculated partition coefficient of these compounds. (* *p* < 0.05, ** *p* < 0.01, two-tailed Mann-Whitney U test). The orange line represents the fit with a Hill function with maximal amplitude 13.7 ± 0.9, midpoint 2.85 ± 0.09, and Hill coefficient 13 ± 5. (**d**) Relation between EC_50_ value and change in anisotropy induced by AP application. The orange line represents the fit with a linear function with a slope of −0.11 ± 0.01 µM (Pearson’s correlation coefficient 0.98, R^2^ = 0.96).

Next, we wanted to confirm the effects of phenolic compounds on membrane properties using DPH. The fluorescence anisotropy of this dye (r) decreases upon membrane fluidization [49,50]. Consistent with this, control experiments showed that the fluidizing agent benzyl alcohol produced a concentration dependent decrease in anisotropy in CHO-TRPA1 cells (Figure 8a). 

Application of APs at concentrations close to the respective EC_50_ for mTRPA1 activation induced similar changes in the anisotropy (Figure 8b). Application of 10 µM phenol or 4-MP did not induce significant changes in anisotropy (*p* = 0.13 and 0.15, respectively, U test). In contrast, APs of longer chains did induce a significant increase, with a tendency to saturation of this effect for 4-HP and 4-OP (Figure 8c). This was reflected in a sigmoidal dependency of the change in anisotropy with the partition coefficient of the compounds. To visualize whether the potency of APs for mTRPA1 activation relates to their ability to decrease membrane fluidity we plotted the EC_50_ values obtained in Ca^2+^ imaging versus the effects of the compounds on the anisotropy parameter (Figure 8d). This shows that rigidity induced by APs correlates with their ability to activate mTRPA1.

### 2.6. Effects of Alkylphenols on Membrane Integrity

**Figure 9:**
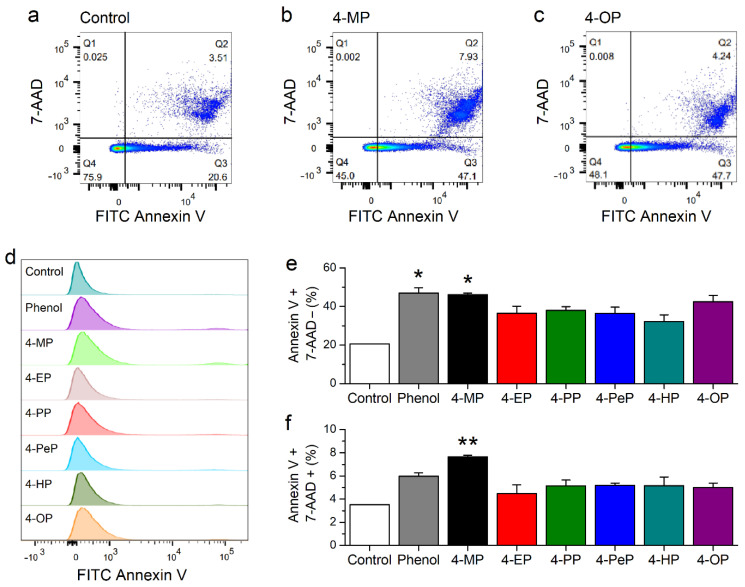
Alkylphenols induced lipid reordering in the plasma membrane. (**a**–**c**) Representative fluorescence-activated cell sorting (FACS) scatter plots of 7-ADD vs Annexin V-FITC obtained in CHO-mTRPA1cells after 10 min in control condition (**a**) and incubation with 1300 μM of 4-MP (**b**) and 5 μM of 4-OP (**c**). The quadrants Q1, Q2, Q3, and Q4 indicate cells labeled Annexin V-negative/7-AAD-positive, Annexin V-positive/7-AAD-positive, Annexin V-positive/7-AAD-negative and Annexin V-negative/7-AAD-negative, respectively. The numbers indicate the respective percentages of total cell population. (**d**) Representative histograms of Q3 (Annexin V-positive, 7-AAD-negative). (**e**,**f**) Average of percentages of Annexin V-positive/7-AAD-negative (**e**) and Annexin V-positive/7-AAD-positive (**f**); * *p* < 0.05, ** *p* < 0.01, *n* = 3, t test.

We further evaluated the effects on APs on plasma membrane properties using a FITC Annexin V Apoptosis Detection Kit with 7-amino-actinomycin D (7-AAD). Annexin V binds to the phosphatidylserine, which is only found on the intracellular leaflet of the plasma membrane in living cells. In conditions in which the membrane asymmetry is lost phosphatidylserine translocate to the external leaflet, allowing the binding of extracellularly applied Annexin V. On the other hand, 7-AAD can bind to DNA only upon disruption of the plasma membrane. Thus, labelling with Annexin V indicates membrane reordering, whereas with 7-AAD reports membrane disruption. We tested the effects of treatment with phenol at 1300 μM or with APs at their respective EC_50_ concentrations for mTRPA1 activation with fluorescence-activated cell sorting (FACS). About 20% of control (untreated) cells were Annexin V-positive/7-AAD-negative (Figure 9a,d,e) and less than 4% were Annexin V-positive/7-AAD-positive (Figure 9a,d,f). The proportion of Annexin V-positive/7-AAD-negative cells was higher in cells exposed to APs than in control cells (Figure 9b–e). The proportion of Annexin V-positive/7-AAD-positive was only higher than in control cells in cells treated with phenol and 4-MP (Figure 9b,d,f).

## 3. Discussion

TRPA1 is arguably the most broadly-tuned chemo-nociceptor known thus far and has key roles in the detection of exogenous noxious chemicals and endogenous mediators signaling tissue damage [11,12,51]. This channel is best known for being activated by reactive electrophiles, via a mechanism that involves covalent modification of cysteine residues in the N-terminus [15,16]. However, TRPA1 can also be activated by a plethora of non-electrophilic compounds, for which the underlying activation mechanisms remain unknown. It is wworth noting that there is no direct evidence for the binding of non-electrophilic agonists to TRPA1 [33,52]. It is conceivable that, as for electrophilic compounds, the detection of non-electrophiles is based on a mechanism that does not rely on specific molecular structures, but rather on a general property of such compounds. This is supported by the consideration that the main function of TRPA1 is not encoding the precise nature of the injurious chemical agent, but to be a primary detector of it, leading to immediate protective responses such as acute pain and inflammation. Another element of support of this idea is that non-electrophilic agonists are very diverse (from small terpenes such as menthol to bacterial lipopolysaccharides (LPS). Thus, it seems unlikely that the channel is activated via classical key-lock interactions, which would require the presence of many distinct binding sites in the channel protein. On the other hand, it is notable that a wide variety of non-electrophilic TRPA1 agonists are able to partition in the plasma membrane, where they are expected to produce local mechanical perturbations.

Previous studies have shown correlations between the potency of non-electrophilic TRPA1 agonists and their lipophilicity, e.g., for thymol and different APs [21], parahydroxybenzoates (parabens) [53], and primary and secondary alcohols, potency positively correlates with lipophilicity [28]. However, to the best of our knowledge, a relation between the abilities to activate TRPA1 and to produce actual changes in physical properties of the membrane has been only shown for bacterial LPS [36].

In this study, we further addressed the hypothesis that the ability of non-electrophiles to activate mTRPA1 is related to their actions on the mechanical properties of the membrane. For this, we studied the effects of alkylated monohydroxyphenolic compounds (APs), as they have a relatively simple structure and allow testing the effects of a series of compounds that differ only in the length of the hydrophobic carbon tail. This represents an advantage over the use of bacterial LPS, as the latter molecules are much more complex and have several differences in both the hydrophilic and the hydrophobic moieties.

We found that all Aps tested are mTRPA1 activators, inducing concentration-dependent and reversible intracellular Ca^2+^ increases, which are abrogated by the TRPA1 inhibitor HC-030031. Although it needs to be confirmed experimentally, these findings raise the possibility that TRPA1 mediates the noxious effects of APs, such as the inhibition of the endocrine function in animals and humans [54] and the repellant, toxic, or antinutritional effects they produce as secondary metabolites used by many plants as defense mechanism against herbivores [2,55].

As for the mechanism underlying AP-induced activation of mTRPA1, it could be envisaged that it can be mediated by the induction of oxidative stress or by the action of electrophilic intermediates [12,43,56,57]. However, we found no evidence of enhanced lipid peroxidation in the presence of APs at concentrations activating mTRPA1. We were further able to discard the possibility that APs acts through such mechanisms, using the argument that activation of TRPA1 by electrophilic agonists occurs via covalent modification of cysteine residues on the channel. Mutating just three of the cysteines (C622S, C642S, and C666S) largely prevents activation by NMM and dramatically reduces sensitivity to other electrophilic compounds such as AITC [15,16,44,58]. We found that APs and the non-electrophilic agonist thymol similarly activated the triple cysteine mutant and the wild type channel. On the other hand, and as expected, the response of the mutant to AITC was less than 50% compared to that of the wild type [44].

Crucially, we found that the potency for mTRPA1 activation of APs strongly correlates with their ability to partition into cellular membranes, but this relation cannot be explained by a simple model based on distinct access of APs to a membrane-buried binding site in mTRPA1. These findings strengthen our original hypothesis that APs activate the channel by inducing mechanical perturbations in the plasma membrane. We reasoned that if this hypothesis was correct, APs should induce changes in the order and fluidity of the plasma membrane. Furthermore, it would be expected that the stronger the effects of APs on the membrane, the stronger would be their agonist action on TRPA1. We tested these ideas by measuring the effects of these compounds on the fluorescent properties of two molecular probes partitioning into different positions in the bilayer, Laurdan and DPH.

A shift towards higher wavelengths of the emission spectrum of Laurdan indicates membrane disordering. Our results show clear readouts in control experiments in which, as expected, benzyl alcohol and heating induced membrane disorder as assessed by a decrease in exGP^340^. On the contrary, all APs increased this parameter in a concentration-dependent manner. This finding is consistent with previous reports showing that benzyl alcohol, phenol, and cresols (4-MP isomers) affect the fluidity of bovine platelets [59,60,61]. However, and confirming our hypothesis, APs with a longer carbon chain were more potent in increasing exGP^340^. In turn, this fits with a previous proposal that, at low concentrations, small aromatic molecules such as benzene accumulate between lipid acyl chains and distribute equally between leaflets with little effect on membrane order [62]. Moreover, the interaction of cresols with the membrane involves hydrogen bond formation between their -OH group and headgroups of lipids at the surface of the bilayer, not perturbing the packing of the phospholipid acyl chains. However, increasing the concentration leads to the partition of these compounds between layers thereby inducing membrane swelling. Once a critical concentration of the compound is reached, the bilayer loses its stability resulting in the membrane separation and finally inducing cell rupture [62]. Our results are in line with these findings, as we found that Laurdan reported an ordering effect of 4-MP at high concentration (800 µM), consistent with the notion that accumulation of these molecules at the bilayer surface restricts penetration of water molecules into the membrane. This is further supported by our FACS data showing that membrane disruption was only induced by phenol and 4-MP when applied at concentrations above 1000 µM.

On the other hand, APs with longer carbon chain are more lipophilic and insert deeper along membrane lipids, with the -OH groups being positioned among the hydrophilic phospholipid headgroups and restricting their translational and rotational degrees of freedom. Accordingly, we found consistently more potent effects of 4-EP, 4-PP, 4-PeP, 4-HP, and 4-OP, in that order, on the spectral shift of Laurdan, which inserts in the hydrophobic core near the lipid headgroups. In the experiments performed with DPH, we found that APs caused an increase in membrane rigidity. However, this effect displays a sigmoidal behavior with the increase in partition coefficient of these compounds, rather than the linear behavior found for the ordering effect reported by Laurdan. According to the DHP readouts, phenol, and 4-MP had no rigidizing effect on the membrane, but APs of longer carbon chains did, and a visible saturation of this effects was observed for 4-HP and 4-OP. These data indicate that phenol and 4-MP do not produce perturbations at the position where DPH partitions in the membrane, which is known to be deeper in the bilayer than for Laurdan. In addition, our data indicates that increasing the hydrophobic chain of the APs from 6 to 8 induced little further enhancement of the rigidity of the membrane at the position of DPH.

Finally, our FACS experiments revealed that phenol and all APs tested have the ability to induced phosphatidylserine relocation to the outer leaflet of the plasma membrane, as evidenced by the higher Annexin V labelling found in cells exposed to these compounds. In addition, phenol and 4-MP showed an ability to disrupt the plasma membrane at high concentration (1300 µM). Although we did not test it, it is expected that APs of longer chains also induce plasma membrane rupture at such concentration.

Thus, all our experiments characterizing the properties of the membrane coincide in that these are altered by APs. Moreover, a key finding is that the concentration range in which APs induce changes in the order, rigidity, and lipid redistribution of the membrane overlaps with the range in which these compounds activate TRPA1. In fact, we found conspicuous exponential relations between the EC_50_ for TRPA1 activation and the increases in membrane ordering (Laurdan’s exGP^340^) and rigidity (DPH fluorescence anisotropy) induced by APs. This further supports the hypothesis that activation of TRPA1 by amphiphilic non-electrophile agonists is related to their ability to induce mechanical perturbations in the plasma membrane. Although the mechanism via which TRPA1 might detect these changes in the lipid bilayer remains to be determined, the structure of the channel protein features multiple grooves that membrane lipids can interact with [63]. This raises the possibility that the insertion of amphiphilic compounds that alter membrane order, rigidity, tension, thickness, or curvature may modify protein-bilayer interactions, which in turn may lead to structural protein adaptations, such as rotations or tilting of helices leading to channel opening [64]. Furthermore, our findings are in line with recent studies showing that TRPA1 is an inherent mechanosensitive ion channel when purified and reconstituted into artificial lipid bilayers [56], similarly to TWIK-related potassium channels (TREK-1 and TREK-2), potassium channel subfamily K member 4 (TRAAK), and Piezo channels that are gated by force-from-lipids [65,66].

Taken together, our results support the hypothesis that mTRPA1 can detect non-electrophilic chemicals by sensing the mechanical alterations they produce in the plasma membrane. This hypothesis is attractive for two reasons. First, it may help explaining how so many structurally unrelated non-electrophiles activate this channel. And second, it entails a mechanism of chemosensation that is not based on the recognition of specific molecular structures, but rather on the detection of compounds that can produce membrane disruption, which is more consistent with the primary role of mTRPA1 as a nociceptive channel. However, more research is needed to verify directly that mTRPA1 can indeed be gated by physical changes in its lipid environment. In the same line, it would be interesting to determine whether the present findings can be reproduced for TRPA1 channels of other species, including humans, and whether they can be observed in native expression systems.

## 4. Materials and Methods

### 4.1. Cell Culture

We used a CHO-K1 stable expression system for mouse TRPA1 (CHO-mTRPA1) [7]. These cells were grown in Dulbecco’s Modified Eagle Medium (DMEM) containing 10% fetal bovine serum, 2% glutamax (Gibco/Invitrogen, Carlsbad, CA, USA), 1% non-essential amino acids (Invitrogen), and 200 µg/mL penicillin/streptomycin at 37 °C and 5% CO_2_. For the series of experiments performed on the mTRPA1-3C/S mutant, HEK293T cells were transiently transfected with mTRPA1 mutant clones in the bicistronic pCAGGS-IRES-GFP vector using TransIT-293 transfection reagent (Mirus Corporation, Madison, WI, USA). Mutations in mTRPA1 were introduced using the QuikChangeTM Site-Directed Mutagenesis Kit (Stratagene, Santa Clara, CA, USA). The nucleotide sequences of the mutants were verified by sequence analysis of the corresponding cDNAs as previously reported [44]. HEK293T cells from the European Collection of Cell Culture (Salisbury, UK) were grown in Dulbecco’s modified Eagle’s medium (DMEM) containing 10% (*v*/*v*) fetal calf serum, 2 mM L-glutamine, 2 U/mL penicillin, and 2 mg/mL streptomycin (Gibco/Invitrogen, Carlsbad, CA, USA) at 37 °C in a humidity-controlled incubator with 10% CO_2_. Cells contamination with mycoplasma species was detected using PlasmoTest-Mycoplasma Detection kit (InvivoGen, Toulouse, France). For most experiments, transfected cells were reseeded after approximately 16 h on 18 mm glass coverslips with thickness of 0.13–0.16 mm (Gerhard Menzel GmbH, Braunschweig, Germany) for ratiometric intracellular Ca^2+^ imaging.

### 4.2. Ratiometric Intracellular Ca^2+^ Imaging

Cells were incubated with 2 µM Fura-2 AM (Biotium, Hayward, CA, USA) for 40 min at 37 °C and 5% CO_2_. Fluorescence was measured with alternating excitation at 340 and 380 nm using a monochromator-based imaging system consisting of an MT-10 illumination system and the Cell^M^ software (Olympus, Tokyo, Japan). All experiments were performed using a Krebs solution containing (in mM): 150 NaCl, 6 KCl, 1 MgCl_2_, 1.5 CaCl_2_, 10 HEPES, 10 glucose, adjusted to pH 7.4 at 25 °C. Fluorescence intensities were corrected for background signal and intracellular Ca^2+^ concentrations were calculated as described previously [67]. Data were analyzed using Origin 9 (OriginLab Corporation, Nothampton, MA, USA).

### 4.3. Electrophysiological Measurements

Automated electrophysiological recordings in CHO-mTRPA1 cells was performed using Patchliner Octo (Nanion Technologies, Munich, Germany) fitted with two EPC-10 quadro amplifiers (HEKA Elektronik GmBH, Lambrecht, Germany) using medium resistance (1.8–3 MΩ) NPC-16 chip (Nanion Technologies). All experiments were performed at 25 °C using the following solutions (in mM): intracellular—10 CsCl, 110 CsF, 20 EGTA, 10 HEPES, 0.3 GTP, 5 ATP, pH 7.2; extracellular—160 NaCl, 4 KCl, 1 MgCl_2_, 2 CaCl_2_, 5 glucose, 10 HEPES, pH 7.4. Cells were held at 0 mV and mTRPA1 currents were elicited by 250 ms steps first to −60 mV and subsequently to +60 mV, at the end of which steady-state currents were measured. Data were collected at a sampling rate of 10 kHz and filtered at 3.33 kHz. Data were acquired using the Patchmaster software (HEKA Elektronik GmBH, Lambrecht, Germany) and analyzed offline using IGOR-Pro (WaveMetrics, Inc. Portland, OR, USA) and Origin 9 (OriginLab Corporation).

### 4.4. Fluorescent Measurements Using Laurdan and DPH

A 0.8 mM stock solution of Laurdan (6-dodecanoyl-N,N-dimethyl-2-naphthylamine) was prepared in methanol (Sigma-Aldrich, Bornem, Belgium). Next, 1.5 µM Laurdan was prepared in Krebs solution (see above) to stain CHO-mTRPA1 cells (1 × 10^6^ cells/mL) for 30 min at 37 °C. After incubation, cells were washed with Krebs, and suspensions of 100 μL were aliquoted into flat-bottom 96-well microtiter plates (Greiner Bio-One, Vilvoorde, Belgium). Steady-state Laurdan fluorescence measurements were performed using a FlexStation III Benchtop Multi-Mode Microplate Reader and the SoftMax Pro Microplate Data Acquisition & Analysis Software (Molecular Devices, Sunnyvale, CA, USA). Laurdan excitation spectra were obtained in the range of 300–420 nm, using both 435 and 490 nm emission wavelengths. Laurdan emission spectra were recorded in the range from 420–550 nm, using both 340 and 410 nm excitation wavelengths. Generalized polarization (GP) values were calculated using emission values at 440 nm and 490 nm, and excitation at 340 nm, according to the formula exGP^340^ = (I_440_ − I_490_)/(I_440_ + I_490_) [45].

A 1 mM stock solution of DPH (Sigma-Aldrich) was prepared in dimethyl sulfoxide (DMSO; Sigma-Aldrich) and a working solution of 10 µM was prepared in Krebs. Cells were stained as described above for Laurdan. DPH anisotropy (r) was monitored with an excitation wavelength of 365 nm and an emission wavelength of 430 nm using a Flexstation III Benchtop Multi-Mode Microplate Reader and the SoftMax Pro Microplate Data Acquisition & Analysis Software (Molecular Devices). A linearly-polarized excitation beam was generated by a vertical polarizer that excites DPH with transition moments aligned parallel to the incident polarization vector. The resulting fluorescence intensities in both parallel (I_VV_) and perpendicular (I_VH_) directions to that of the excitation beam were recorded and the fluorescence anisotropy can be calculated by: r = (I_VV_ − G∙I_VH_)/(I_VV_ + 2G∙I_VH_), where G = I_HV_/I_HH_. The relation between anisotropy (r) and polarization (P) was determined as P = 3r/(2 + r) and the membrane fluidity was calculated as the inverse of fluorescence polarization (1/P) [68].

### 4.5. Fluorescence-Activated Cell Sorting

CHO-mTRPA1 cells were treated with APs for 10 min and stained with APC Annexin V Apoptosis Detection Kit with 7-AAD (BioLegend, San Diego, CA, USA) according to the manufacturer protocol. Percentages of labeled cells were determined by performing flow cytometry on a Canto II HTS cytometer (BD Biosciences, San Jose, CA, USA) in triplicates. Data analysis was performed with FlowJo software (v10.5.3, FlowJo LLC, Ashland, OR, USA).

### 4.6. Lipid Peroxidation Assay

CHO-mTRPA1 cells were seeded into flat-bottom 96-well microtiter plates (Greiner Bio-One) and grown to confluency. Cells were treated with APs or H_2_O_2_ (300 μM) in PBS for 10 and 30 min at 37 °C in a cell culture incubator. The compounds were removed and BODIPY 581/591 C11 (Thermo Fisher Scientific, Eugene, OR, USA, 10 μm final concentration) was added. After 30 min in culture, cells were washed and resuspended in PBS and the fluorescence of C11 BODIPY was monitored with excitation/emission of 581/591 nm for the reduced dye, and excitation/emission of 488/510 nm for the oxidized dye using a Flexstation III Benchtop Multi-Mode Microplate Reader and the SoftMax Pro Microplate Data Acquisition & Analysis Software (Molecular Devices). The ratio of the emission fluorescence intensities at 591 nm to 510 nm gives the read-out for lipid peroxidation in cells.

### 4.7. Data and Statistical Analysis

Data are presented as mean ± s.e.m. using Origin 9.0 (OriginLab Corporation). If not stated otherwise the non-parametric Mann-Whitney test was used to assess statistical significance using GraphPad Prism software. In the figures, the asterisks indicate the level of statistically significant differences (*, *p* < 0.05 and **, *p* < 0.01).

### 4.8. Nomenclature of Targets and Ligands

Key protein targets and ligands in this article are hyperlinked to corresponding entries in http://www.guidetopharmacology.org (accessed on 28 January 2021), the common portal for data from the International Union of Basic and Clinical Pharmacology (IUPHAR) and the British Pharmacological Society (BPS). Guide to PHARMACOLOGY [69], and are permanently archived in the Concise Guide to PHARMACOLOGY 2017/18 [70].

### 4.9. Calculation of Partition Coefficients

JChem for Office was used for chemical database access, structure-based property calculation, search and reporting, JChem for Office 21.2.0.797, 2021, ChemAxon (http://www.chemaxon.com, accessed on 28 January 2021). LogP of each molecule was calculated based on the consensus model, that uses ChemAxon and Klopmans models and the PhysProp database.

## Figures and Tables

**Table 1:** List of reagents used in the study, structure, partition coefficient (logP). The purity is above 97% for all compounds.

Name	Short Name	Structure	logP
4-methylphenol	4-MP	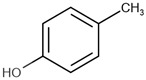	2.18
4-ethylphenol	4-EP	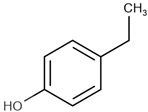	2.63
4-propylphenol	4-PP	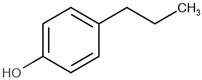	3.07
4-pentylphenol	4-PeP	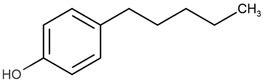	3.96
4-heptylphenol	4-HP	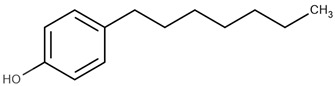	4.85
4-octylphenol	4-OP	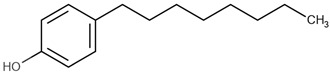	5.30

## Data Availability

Data is contained within the article.
